# Nose-to-brain delivery of a SOD1-stabilizing small molecule ameliorates pathology in an ALS mouse model

**DOI:** 10.1016/j.neurot.2026.e00904

**Published:** 2026-04-10

**Authors:** Ranjithkumar Dhandapani, Shamchal Bakavayev, Anna Armoza, Marina Bersudsky, Arina Shlifer, Galit Yehezkel, Alexandra Tsitrina, Zeev Barak, Amnon C. Sintov, Stanislav Engel

**Affiliations:** a The Department of Clinical Biochemistry and Pharmacology, Faculty of Health Sciences, Ben-Gurion University of the Negev, Beer-Sheva, 84105, Israel; b The Shraga Segal Department of Microbiology, Immunology and Genetics, Faculty of Health Sciences, Ben-Gurion University of the Negev, Beer-Sheva, 84105, Israel; c The Department of Life Sciences, Faculty of Natural Sciences, Ben-Gurion University of the Negev, Beer-Sheva, 84105, Israel; d Katz Institute for Nanoscale Science and Technology, Ben-Gurion University of the Negev, Beer-Sheva, 84105, Israel; e The Department of Biomedical Engineering, Faculty of Engineering Science, Ben-Gurion University of the Negev, Beer-Sheva, 84105, Israel; f The Zlotowski Center for Neuroscience, Ben-Gurion University of the Negev, Beer-Sheva, 84105, Israel

**Keywords:** Small-molecule compounds, ALS, SOD1, Misfolding, Nose-to-brain CNS delivery, Virtual screen

## Abstract

Exposure of a pathogenic β6/β7 loop neo-epitope has been proposed to contribute to the pathogenesis of misfolded Cu/Zn superoxide dismutase (SOD1) in amyotrophic lateral sclerosis (ALS) by mediating early events in its noxious structural transformation and prion-like activity. Antibody-mediated blockade of this epitope was shown to ameliorate disease phenotype in an ALS animal model. Here, as an alternative strategy, we sought to block this epitope using a small molecule designed to occupy the inter-subunit cavity framed by the two β6/β7 loops. Using a structure-based virtual screen targeting this cavity, we identified a small molecule, N-[3-(3-methylimidazo[2,1-b][1,3]thiazol-6-yl)phenyl]-4-sulfamoylbenzamide (C7), that preferentially bound the native-like conformation of SOD1, reduced β6/β7 loop epitope accessibility, and inhibited irreversible apo-SOD1 misfolding in vitro. Delivered to presymptomatic hSOD1^G93A^ mice via a nanoparticle-based nose-to-brain delivery system, C7 significantly delayed the onset of motor abnormalities and modestly extended survival. At disease onset, spinal cord analysis revealed reduced misfolded SOD1 inclusions and attenuated astro- and microgliosis. Analysis of C7 concentrations in combined brain and spinal cord tissue indicated rapid but saturable nose-to-CNS uptake and slow clearance. Our findings demonstrate that targeting the surface cavity shaped by the β6/β7 loops of SOD1 with a reversibly-binding small molecule can ameliorate ALS-like disease *in vivo*, potentially by counteracting early misfolding events and/or limiting prion-like propagation of molecular pathology. However, saturable nose-to-CNS uptake of C7 restricts CNS exposure and likely constrains therapeutic efficacy, underscoring the need to define the rate-limiting pharmacokinetic step and to optimize the nanoparticle formulation and/or physicochemical properties of the C7 scaffold.

## Introduction

Superoxide dismutase is a major antioxidant enzyme responsible for the scavenging of superoxide anions in human body, and in its native conformation, a constitutive homodimer characterized by extraordinarily structural stability with an estimated half life of more than hundred years [1]. The enzymatic and structural properties of SOD1 depend on the presence of metal cofactors, of which zinc provides major stabilizing effect on the SOD1 structure, while copper is essential for its enzymatic activity. The loss of the stabilizing metal cofactors results in the destabilization of the dimer interface and decreases the estimated half life of the protein from years to hours [1,2].

Numerous mutations in the SOD1 gene have been found to associate with the increased risk of ALS, a terminal neurodegenerative disorder, which affects motor neurons in brain and spinal cord [3]. SOD1 pathogenesis in ALS has been attributed to neurotoxic properties the protein acquires upon the loss of its stabilizing metal cofactors, the process facilitated by mutations, but which also could be promoted by post-translational modifications of the wild type protein, such as oxidation [4,5]. Metal deficient SOD1 undergoes a gradual structural transition, referred to as misfolding, whose ultimate outcome is the formation of metal-free amyloid like aggregates in the affected cells of brain and spinal cord [[6], [7], [8]]. The growing body of evidence attributes the neurotoxic properties of SOD1 to low-molecular weight soluble species of misfolded SOD1, potentially monomeric or oligomeric, whose mechanism of toxicity remains obscure [9,10]. One possibility is the acquired ability of misfolded SOD1 to aberrantly interact with a variety of structurally diverse proteins, including its own species, thus interfering with their normal function [11,12]. The anomalous protein-protein interactions (PPI) of a homological type are accountable for the prion-like activity of misfolded SOD1, responsible for the cell-to-cell propagation of the misfolding signal, part of the SOD1 pathogenic mechanism in ALS [13,14].

We have previously hypothesized that exposure of a pathogenic β6/β7 loop epitope, which is concealed in the native SOD1 dimer but being revealed in the metal-deficient protein, is implicated in the early steps of noxious SOD1 structural transformation [15]. Once exposed, the β6/β7 loop epitope engages in aberrant protein–protein interactions with a surface cavity shaped by the two parallel β6/β7 loops of an intact SOD1 dimer, resulting in the formation of an aberrant trimeric complex in which destabilization of the dimer interface promotes further SOD1 misfolding [15]. A monoclonal antibody developed against the β6/β7 loop epitope (SE21 mAb) recognized demetallated (apo) forms of SOD1, both WT and ALS mutants, but not intact metalated (holo) SOD1 [16]. The SE21 expressed in the CNS of ALS mice transgenic for a mutated version of human SOD1 as a scFv intrabody, strongly delayed disease onset and extended survival [17]. These results support the notion of the importance of the exposed the β6/β7 loop epitope to SOD1 pathogenesis in ALS and suggest that its blocking may constitute a viable therapeutic strategy for ALS.

Here, we explored an alternative to a blocking-antibody strategy to prevent the formation of early aberrant complexes by misfolded SOD1, based on the use of a small-molecule compound designed to fit into the surface cavity formed by the two parallel β6/β7 loops of the SOD1 dimer. Occupancy of this cavity in the intact SOD1 dimer is expected to interfere with its engagement by misfolded SOD1 and thereby prevent formation of the aberrant trimeric complex described above [15]. We virtually screened a library of drug-like compounds for molecules capable of fitting into the surface pocket delineated by the two β6/β7 loops of native SOD1. Experimental testing of hit compounds for their ability to inhibit binding of the SE21 mAb to demetallated SOD1 led to the identification of a compound, C7 (N-[3-(3-methylimidazo[2,1-b][1,3]thiazol-6-yl)phenyl]-4-sulfamoylbenzamide), which exhibited a stabilizing effect on SOD1 structure. CNS delivery of C7 via a direct nose-to-brain nanoparticle-based system ameliorated disease phenotype in an aggressive ALS mouse model transgenic for the human SOD1^G93A^ variant, significantly delaying disease onset, extending survival, and suppressing neuroinflammation.

## Materials and Methods

### Virtual screen

The structure-based virtual screen (VS) was carried out using the Molecular Operating Environment (MOE) software (Chemical Computing Group Inc.) and the crystal structure of metalated SOD1^WT^ dimer (PDB: 1HL5) as targeted structure. The structure was prepared using an automated (QuickPrep) procedure implemented in MOE, and the final structure was analyzed using the Site Finder module, which ranks potential small-molecule binding sites according to the Propensity for Ligand Binding (PLB) score, based on the amino acid composition of the pocket [18]. The surface cavity shaped by the two parallel β6/β7 loops of SOD1 dimer (SITE 1, Supplementary Fig. S1A) was ranked first with the PLB score of 3.14. This cavity was used to formulate a 3D structure-based pharmacophore, whose six features (two proton donors and 4 proton acceptors) were contributed by both subunits of the dimer (Supplementary Fig. S1A).

The library of two millions of commercially available drug-like compounds from MOE collection and ZINC database [19] were virtually screened using a multistep MOE docking protocol, comprising the following steps: (**a**) The compounds were docked into SITE 1 using the 3D pharmacophore as placement method and London dG scoring function, (**b**) The binding poses of the 10% percent of top ranked compounds from step [1] were refined using fixed side chains (Rigid Receptor protocol) and flexible ligands with pharmacophore restraints, and rescored using GBVI/WSA dG scoring function, (**c**) The binding poses of 10% percent of the top ranked compounds from step (b) were refined using Induced Fit protocol with side chains tethered and ligand flexible with pharmacophore restraints, and rescored using GBVI/WSA dG scoring function, (**d**) The binding poses of 10% percent of the top ranked compounds from step (c) were refined using Induced Fit protocol with side chains tethered and ligand flexible without pharmacophore restraints, and rescored using GBVI/WSA dG and London dG scoring functions. One thousand compounds top ranked with both GBVI/WSA dG and London dG scoring functions (consensus) from step (d) were used to calculate a diverse subset of 100 compounds using MACCS Structural Keys fingerprints and Tanimoto Coefficient as similarity matrix, which was then purchased from MolPort SIA (EU) for experimental testing.

### Protein preparation

Human SOD1^WT^ and SOD1^G93A^ were produced recombinantly in *E. coli* BL21 cells cultured in the absence of supplemental Cu^2+^ and Zn^2+^ ions. Proteins were purified to homogeneity under non-denaturing conditions following previously reported procedure [16]. Conversion of the purified proteins to their demetallated (apo) forms, as well as preparation of fully metalated (holo) SOD1, was carried out according to established protocols [16]. Protein concentrations were quantified using the Bradford method with bovine serum albumin as the calibration standard. For biolayer interferometry (BLI) experiments, purified proteins were covalently biotinylated (at ∼1:1 biotin-to-protein molar ratio) using the EZ-Link™ NHS-PEG4 Biotinylation Kit (Thermo Fisher Scientific Inc.) in accordance with the manufacturer's guidelines.

### C7 inhibition of SE21 mAb binding to apo-SOD1 measured by ELISA

ELISA 96-well plates (Nunc MaxiSorp, Thermo Fisher Scientific) were coated with 0.5 μg per well of apo-SOD1 (WT or G93A variant) in 100 μL PBS. After incubation for 30 min at RT., plates were transferred to 4 °C and incubated overnight. The following day, plates were washed twice with washing buffer (PBS containing 0.05% Tween-20, PBST) and blocked with 200 μL per well of 1% BSA in PBS for 1 h at 37 °C. The blocking solution was removed, and plates were washed twice with PBST. Plates were then incubated with the indicated concentrations of C7 prepared in PBS containing 2% DMSO. After incubation for 1 h at room temperature, 50 μL of PBS containing either rat monoclonal SE21 antibody [16] (15 ng per well) or sheep polyclonal anti-SOD1 antibody (NB200-600, Novus Biologicals, 1:1000) was added. Plates were incubated for 30 min at RT and then washed four times with PBST. HRP-conjugated secondary antibodies diluted in blocking buffer (100 μL per well) were then added as follows: goat anti-rat IgG (A18871, Thermo Fisher Scientific; 1:10,000) for SE21 mAb detection, or rabbit anti-sheep IgG (313-035-045, Jackson ImmunoResearch; 1:20,000) for polyclonal anti-SOD1 detection. Plates were incubated for 1 h at 37 °C, washed four times with PBST, and developed using 100 μL per well of TMB substrate solution (KPL). Color development was allowed to proceed at room temperature in the dark and was terminated by addition of 100 μL TMB stop solution (KPL). Absorbance was measured at 450 nm using an Infinite® 200 PRO microplate reader (Tecan).

### C7 binding to SOD1 measured by microscale thermophoresis (MST)

The analysis was performed using a Monolith® NT.115 Pico instrument (NanoTemper Technologies GmbH, Germany), employing a 1480 nm infrared laser to generate a temperature gradient and a 488 nm excitation light source coupled to a 535/50 band-pass filter for fluorescence detection. Protein labeling was carried out using the Monolith NT™ Protein Labeling Kit BLUE–NHS (Cat. #L003, NanoTemper Technologies) according to the manufacturer's protocol with minor modifications. Recombinant SOD1 proteins (either holo-SOD1^WT^ or apo-SOD1^G93A^) were adjusted to 20 μM in PBS, and 100 μL aliquots were subjected to buffer exchange into labeling buffer using the provided Column A. Proteins were then incubated with NT™ Blue dye at a final concentration of 40 μM, corresponding to an approximate 2:1 dye-to-protein molar ratio, for 30 min at room temperature in the dark. Excess unreacted dye was removed using Column B, and proteins were eluted with PBS containing 0.05% Tween-20 (PBST) as analysis buffer. Eluted fractions were collected in ∼150 μL aliquots (total volume ∼1.2 mL). Aliquots of the eluted fractions, diluted 4–10-fold, were loaded into standard capillaries (NanoTemper Technologies) and measured on the Monolith NT.115 to assess fluorescence intensity and identify the fraction with optimal labeling efficiency and signal quality. Using the selected fraction, optimal measurement conditions (including protein concentration, LED power, and signal-to-noise ratio) were calibrated according to NanoTemper guidelines. Labeled protein samples were stored at 4 °C until use.

For microscale thermophoresis (MST) measurements, 10 μL aliquots of the labeled protein at the optimized working dilution were mixed with 10 μL of PBST containing 2% DMSO and a 2-fold dilution series of compound C7, yielding final C7 concentrations ranging from 50 μM to 6 nM. The calculated final protein concentration in the MST assay was in the low nanomolar range (100–200 nM), consistent with recommended MST conditions. After incubation for 2 h at RT in the dark, samples were loaded into capillaries and measured. Data were analyzed using NT Analysis software version 1.5.41 (NanoTemper Technologies) and GraphPad Prism 6.

### C7 binding to SOD1 measured by biolayer interferometry (BLI)

Biotinylated target protein (40 μg/mL in PBS) was immobilized onto Octet® high-capacity Super Streptavidin (SSA) biosensors (Sartorius Lab Instruments GmbH & Co.), followed by quenching of residual streptavidin binding sites with 10 μg/mL EZ-Link™ Biocytin (Thermo Fisher Scientific Inc.). Binding kinetics of compound C7 were measured using an Octet® BLI R8 instrument (Sartorius Lab Instruments GmbH & Co.) at room temperature in assay buffer (PBS, 2% DMSO), using increasing concentrations of C7. In parallel, reference SSA biosensors quenched with biocytin were exposed to the same C7 concentrations, and the resulting sensorgrams were used for background subtraction. Data were processed and analyzed using Octet® Analysis Studio software (Sartorius Lab Instruments GmbH & Co.).

### Inhibition of SOD1 misfolding by C7

The kinetics of apo-SOD1^WT^ irreversible structural transition was analyzed as previously described [16]. Prior to experiments, the protein-containing solutions were passed through a 0.22 μm PVDF syringe filter (Millex-GV, Millipore) to remove pre-existing aggregates. Recombinant apo-SOD1^WT^ (50 μM) was incubated at 37 °C under misfolding-promoting conditions (20 mM HEPES buffer, pH 7.4, 100 mM NaCl and 1 mM TCEP, with continuous agitation (800 rpm)). Aliquots were collected at the indicated time points and incubated with 100 μM ZnCl_2_ for 2 h at RT to stabilize residual native-like conformers. Following Zn^2+^ reconstitution, samples (0.5 μg protein per well) were immobilized onto high-binding 96-well plates (Nunc MaxiSorp, Thermo Fisher Scientific) by incubation for 2 h at RT in 100 μL of 20 mM HEPES buffer, pH 7.4, 100 mM NaCl. Plates were rinsed with imidazole-based washing buffer (KPL) and blocked with 1% (w/v) BSA in PBS for 1 h at RT. Misfolded SOD1 species were detected by incubation with the conformation-selective monoclonal antibody SE21 (rat; RRID: AB_2827827; 0.07 μg per well) diluted in 1% (w/v) BSA in PBS for 1 h at room temperature. After washing, plates were incubated with HRP-conjugated goat anti-rat secondary antibody (1:10,000; ThermoFisher Scientific; RRID: AB_2535648) for 1 h at room temperature. Colorimetric detection was performed using TMB Microwell Peroxidase Substrate (KPL), and the reaction was terminated with TMB stop solution (KPL). Absorbance was measured at 450 nm using an Infinite® 200 PRO microplate reader (Tecan).

### Nanoparticulate system preparation

C7-loaded amylolipid nanovesicles (C7-ALNs) were formulated as modified starch-lipid hybrid nanoparticles for intranasal delivery, as described [20]. Briefly, ALNs consist of a solid lipid core encapsulated within a divanillin-crosslinked starch shell. The formulations were prepared by using a microemulsion as the precursor based on polyoxyl 40 hydrogenated castor oil, Theobroma oil, tetraglycol and glyceryl oleate (0.4:1.0:3.4:1.9). Gelatinized corn starch (4%) was dispersed with divanillin and C7 in microemulsion under stirring for 1 h, then the pH was adjusted to 6 and the system was diluted appropriately with water. The concentration of C7 in the final ALN dispersion was 0.14 mg/mL, which is accounted for 1.4 μg dose per animal (5 μl volume per nostril × 2) (NP Formulation 1). To increase the dose, a modified lipid nanoparticulate system (LNP) was prepared by just replacing starch with alginic acid. The concentration of C7 in the final LNP dispersion was 3.0 mg/mL, which is accounted for 30 μg dose per animal (5 μl volume per nostril × 2) (NP Formulation 2).

### Animal studies

Transgenic mice carrying the human SOD1^G93A^ mutation (B6.Cg-Tg(SOD1∗G93A)1Gur/J; RRID: IMSR_JAX:004435) were originally obtained from The Jackson Laboratory (USA) and maintained on a congenic C57BL/6J background (Strain #000664) to minimize background-related genetic variability. Genotype verification was performed by PCR analysis of tail biopsy DNA following the supplier's recommended protocol, with non-transgenic littermates serving as negative controls. All experimental animals were derived from an established in-house breeding colony housed at the Ben-Gurion University animal facility and maintained under standardized husbandry conditions.

Animals were kept in a specific pathogen-free environment with controlled temperature and humidity and a 12 h light/dark cycle, with unrestricted access to standard chow and water. Following the emergence of disease symptoms, supportive care was provided, including wetted food pellets, water bottles with extended spouts, and assisted bladder expression twice daily when required. Animal health and welfare were monitored routinely according to institutional guidelines. No animals exhibited infections or unrelated health abnormalities prior to study initiation, and none had been used in previous experimental procedures. All procedures were conducted in accordance with protocols approved by the Ben-Gurion University Institutional Animal Care and Use Committee (IACUC protocol # BGU322-05-2024E).

Sample size was determined in advance by power analysis for a two-group comparison (treatment versus control, 1:1 allocation). Assuming a coefficient of variation of 20%, a treatment-induced delay in disease onset of 20%, a significance level of α = 0.05, and statistical power of 80% (β = 0.20), the calculated minimum group size was 16 mice per arm. To enable biochemical and histological analyses at defined endpoints, an additional eight animals per group were included.

Eligibility criteria were specified prior to study commencement. Male and female transgenic mice aged 25–28 days and weighing 10–15 g were enrolled sequentially and randomly assigned to experimental groups. Animals were required to be clinically normal and free of visible injury, infection, or distress at baseline and throughout the experimental period. Mice developing health conditions unrelated to disease progression (including >20% weight loss, infection, or abnormal behavior) were excluded in accordance with animal welfare regulations. No animals were excluded from ongoing experiments, and no technical or analytical outliers were identified during data analysis. All procedures—including behavioral assessment, tissue processing, image quantification, and statistical analysis—were conducted under blinded conditions until completion of the primary analysis.

Prior to intranasal (IN) delivery experiments, animals were acclimated to manual restraint to minimize stress. For intranasal administration, each mouse was gently restrained in an upright position, with the body supported and the back of the neck lightly grasped to maintain normal respiration. A total volume of 10 μL was administered per dosing event, delivered as 5 μL into each nostril using a calibrated micropipette and applied as two sequential drops per nostril (∼2.5 μL each), with an interval between drops to allow inhalation. In cases of accidental sneezing, the expelled dose was reapplied. Administrations were performed at the same time each day throughout the experiment to maintain consistency and minimize circadian or handling-related variability. Anesthesia was not used in order to preserve physiological respiration, natural olfactory airflow, and normal mucociliary clearance. Following administration, mice were monitored for signs of local irritation, redness, swelling, or allergic reactions around the nostrils; no such effects were observed during the dosing period. Prior to the main experiments, a short-term (2-week) preliminary toxicity study involving IN administration of C7 was conducted and revealed no observable behavioral signs of toxicity.

For the principal efficacy experiment using NP Formulation 2, treatment was initiated at 25–28 days of age and continued once daily until the experimental endpoint. Kaplan–Meier analyses were performed separately by sex. The male cohort consisted of 12 C7-treated mice and 11 control mice (empty-NP); the female cohort consisted of 10 C7-treated mice and 10 control mice. In the supplementary low-dose efficacy experiment with Formulation 1, cohort sizes were n = 12 for C7-treated mice and n = 10 for empty-NP controls, with males and females equally represented in each cohort.

### Behavioral assessment

Disease progression was monitored twice weekly by body weight, grip strength, and neurological scoring. Neurological function was assessed using the NeuroScore (NS) system [21] during tail suspension, free ambulation, and, at later stages, after placement of the animal on its side. Scores were assigned as follows: NS0, normal hindlimb extension during tail suspension and normal gait; NS1, partial hindlimb collapse or tremor during tail suspension with preserved gait; NS2, complete hindlimb collapse during suspension with impaired locomotion and foot dragging; NS3, rigid hindlimb paralysis or minimal joint movement with limited forward motion and preserved righting within 20 s; NS4, humane endpoint, defined by failure to self-right within 20 s. Disease onset was defined as the first occurrence of NeuroScore 1. Survival was defined as the age at which animals reached NeuroScore 3 (progression to paralysis).

Forelimb and hindlimb grip strength [22] were measured using a Chatillon DFE II digital force gauge (Ametek). For each trial, mice were allowed to grasp a horizontal wire grid and were then gently pulled backward by the tail until grip release. Three consecutive measurements were obtained for each configuration, and the mean peak force value was used for analysis.

### Measurement of C7 concentration in the CNS by high-performance liquid chromatography (HPLC)

Mice were euthanized at defined post-administration time points (1, 3, or 6 h). From each mouse, the entire brain and spinal cord were removed without perfusion, briefly rinsed by immersion in 50 mL PBS with gentle stirring, gently blotted dry, and weighed. Samples were then frozen at −80 °C and vacuum-dried. Dry weights were recorded, and samples were stored at −80 °C until analysis.

Freeze-dried combined brain and spinal cord tissue was ground and extracted with methanol, followed by centrifugation. Aliquots (20 μL) of the resulting supernatants were injected into an HPLC system (1260 Infinity II, Agilent Technologies Inc., Santa Clara, CA) equipped with a C18 column maintained at 25 °C, an autosampler, and a diode array detector. Chromatographic separation was performed using an acetonitrile/0.25% formic acid mobile phase (60:40, v/v) at a flow rate of 1 mL/min. Quantification of C7 was carried out at 236 nm using a standard calibration curve, with a limit of quantitation of 10 ng/mL.

### Immunohistochemistry

For histological immunofluorescence (IF) analyses, lumbar spinal cords were collected from female mice only (n = 5 per group) at 90 days of age, a time point corresponding to the onset of reproducible motor impairment (NeuroScore 1) in control mice. Mice were anesthetized with 1.5–3% isoflurane and transcardially perfused first with 50 mL of ice-cold PBS containing 10 U/mL heparin (Sigma-Aldrich) delivered at 5 mL/min, followed by 20 mL of 4% paraformaldehyde (PFA) in PBS at RT., delivered at the same flow rate, for tissue fixation. Following perfusion, spinal cords were dissected and post-fixed overnight in 4% PFA at 4 °C. Tissues were then cryoprotected by sequential incubation in 10%, 20%, and 30% (w/v) sucrose in PBS until fully equilibrated, as indicated by tissue sinking. Cryoprotected cords were embedded in OCT compound (Tissue-Plus, Scigen), rapidly frozen on dry ice, and sectioned coronally at 50 μm using a cryostat.

Free-floating sections were washed three times in PBS and incubated for 24 h at RT in blocking solution (10% serum from the secondary antibody hosts, 0.4% Triton X-100, 0.05% sodium azide in PBS). Sections were then incubated for 48 h at 4 °C with primary antibodies diluted in antibody solution (10% host serum, 0.05% Triton X-100, 0.05% sodium azide in PBS). After three washes in PBS containing 0.05% Triton X-100, sections were incubated for 4 h at room temperature with fluorophore-conjugated secondary antibodies prepared in antibody solution. Sections were then mounted on glass slides, air-dried, and coverslipped using DAPI Fluoromount-G® (Southern Biotech) antifade mounting medium. When mouse primary antibodies were used, a mouse-on-mouse (M.O.M.) blocking step (ScyTek) was incorporated following the initial blocking phase and applied for 24 h at 4 °C to suppress background originating from endogenous immunoglobulins.

Primary antibodies were: misfolded human SOD1 (mouse, MédiMabs, clone B8H10, CAT# MM-0070-P, RRID: AB_2909641, 1:200), choline acetyltransferase (ChAT; rabbit, GeneTex, clone N1N3, CAT# GTX113164, RRID: AB_1949973, 1:200), glial fibrillary acidic protein (GFAP; rabbit, Cell Signaling Technology, clone E4L7M, CAT# 80788, RRID: AB_2799963, 1:200), and ionized calcium-binding adaptor molecule 1 (Iba1; rabbit, FUJIFILM Wako, CAT# 019-19741, RRID: AB_839504, 1:1000). Secondary antibodies were: goat anti-mouse Alexa Fluor 647 (Thermo Fisher Scientific, CAT# A-21235, RRID: AB_2535804, 1:2000) and goat anti-rabbit Alexa Fluor 555 (Thermo Fisher Scientific, CAT# A-21428, RRID: AB_2535849, 1:2000).

### Image acquisition and analysis

Lumbar spinal cord sections were imaged on a Zeiss LSM880 confocal microscope equipped with an Airyscan detector using 20 × and 40 × (oil immersion) objectives. All images were acquired under identical optical conditions, with laser excitation power and detector gain maintained constant across experimental groups. Image processing and quantitative analyses were performed using ImageJ/Fiji (NIH, USA). Image acquisition and analysis were conducted in a blinded manner. Cholinergic ChAT-positive motor-neuron quantification was performed as a profile-based approach to enable comparative assessment between experimental groups and not by unbiased stereological optical fractionation. For each animal, five lumbar spinal-cord coronal sections spanning the L3–L5 region were analyzed at 3-section intervals, separated by approximately 150 μm, to minimize repeated sampling of the same neuron in adjacent sections. Within each section, ChAT-positive neuronal profiles in the ventral horn were identified using predefined intensity and size/shape thresholds in ImageJ/Fiji and then manually verified by an investigator to blinded group allocation. Only ChAT-positive cells meeting the following criteria were counted: large ventral-horn soma, polygonal morphology, and a clearly identifiable nucleus. Counts from the five sections were averaged to obtain one value per animal.

For quantification of misfolded SOD1, GFAP, and Iba1 immunoreactivity, 16-bit grayscale images were analyzed. The gray-matter “butterfly” was manually delineated as the region of interest (ROI), and mean pixel intensity within the ROI was measured. For each marker, five sections spanning the L3–L5 levels of the spinal cord were analyzed per animal and averaged, with five animals included in each group.

### Statistical analyses

Statistical analyses were performed using GraphPad Prism 6. Data are presented as mean ± SEM unless otherwise indicated; HPLC data are presented as mean ± SD. Kaplan–Meier analyses of disease onset and survival were compared using the log-rank (Mantel–Cox) test, and hazard ratios with 95% confidence intervals were calculated using the Mantel–Haenszel method. Comparisons between two groups in histological and selected in vitro assays were performed using unpaired two-tailed Student's *t*-test, as specified in the corresponding figure legends. MST binding curves were analyzed by nonlinear regression using a three-parameter logistic model. BLI sensorgrams were analyzed using a 1:1 Langmuir binding model together with steady-state fitting, as described above. A P value < 0.05 was considered statistically significant. Where applicable, sex was analyzed separately in survival and behavioral experiments.

## Results and Discussion

### Virtual screen

**Fig. 1:**
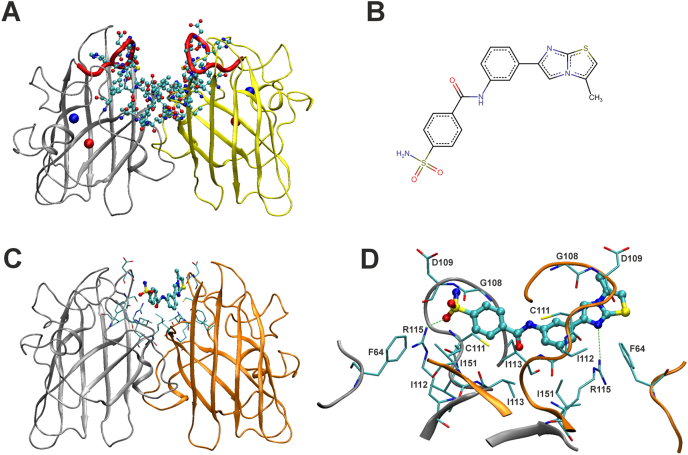
**Identification of C7 as a small molecule predicted to bind the β6/β7 loop surface cavity of the SOD1 dimer.** (**A**) Structure of the intact holo-SOD1^WT^ dimer (PDB: 1HL5) showing, in ball-and-stick representation, the surface cavity formed by the two parallel β6/β7 loops (SITE 1; see Methods) used to formulate the 3D pharmacophore hypothesis employed in virtual screening (VS). The sequences corresponding to the SE21 mAb binding epitope [16] are highlighted in red. Blue and red spheres correspond to zinc and copper ions, respectively. (**B**) Chemical structure of C7. (**C**) Overall view of the C7–SOD1 dimer complex as predicted by VS docking, with C7 fitted into the surface cavity delineated in (A). (**D**) Detailed view of the C7–SOD1 interaction network, showing that residues from both SOD1 subunits contribute to stabilization of C7 within the binding site. Hydrogen bonds between C7 and Arg115 residues from the two SOD1 subunits are indicated.

We employed a structure-based virtual screen (VS) to identify a compound expected to prevent the formation of the aberrant trimeric complex formed by misfolded SOD1 (see the Introduction and Ref. [15]) by fitting into the cleft between the two parallel β6/β7 loops in the intact SOD1 dimer (Fig. 1A). To accomplish this, we constructed a 3D pharmacophore of the surface cavity present in the holo-SOD1^WT^ dimer (PDB: 1HL5) (Supplementary Fig. S1A) and used it as a primary placement site for compound docking. A library of two million commercially available drug-like compounds was virtually screened as described in the Methods, and one hundred highly ranked, structurally diverse compounds from the hit list were purchased and experimentally tested.

### C7 compound preferentially binds a native-like conformation of SOD1

The mapped SE21 mAb epitope exposed in demetallated (apo) SOD1 [16] partially overlaps with the surface cavity in the intact SOD1 dimer that we used for compound docking (Fig. 1A). We therefore assumed that binding of a compound to this cavity would sterically interfere with SE21 mAb binding to apo-SOD1. Moreover, SE21 mAb binding to demetallated SOD1 could also be prevented if compound binding exerts a stabilizing effect on SOD1 structure analogous to that conferred by metal cofactors, since exposure of the β6/β7 loop epitope is thermodynamically coupled to metal cofactor loss [16]. Collectively, SE21 mAb binding can thus be used to monitor compound interaction with demetallated SOD1 that results in masking of the pathogenic β6/β7 loop epitope.

**Fig. 2:**
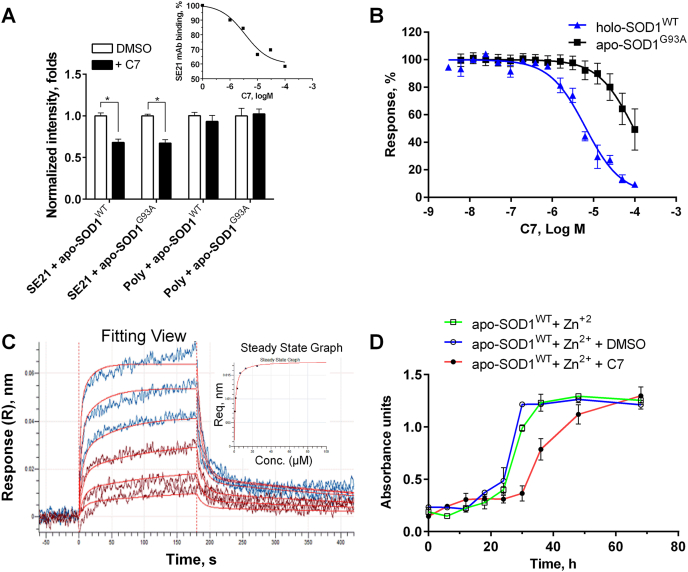
**The C7 compound preferentially binds and stabilizes a native-like conformation of SOD1.** (**A**) C7 (50 μM) inhibits binding of SE21 mAbs to apo-SOD1^WT^ and the fALS-associated SOD1 mutant G93A, measured by ELISA. *Inset:* SE21 mAb binding to apo-SOD1^WT^ in the presence of increasing concentrations of C7. Poly = polyclonal anti-SOD1 Ab. Results are expressed as mean ± SD of assays performed in triplicate in a representative experiment. Statistical significance between groups was assessed using an unpaired parametric *t*-test; ∗p < 0.05. (**B**) MST analysis of C7 binding to holo-SOD1^WT^ and apo-SOD1^G93A^. Curves represent nonlinear regression analyses using a three-parameter logistic function. Results are expressed as mean ± SE from at least three independent experiments. (**C**) Representative biolayer interferometry (BLI) sensorgrams showing concentration-dependent binding of C7 to biotinylated, immobilized holo-SOD1^WT^. Experimental traces are shown together with global fits to a 1:1 Langmuir binding model. The inset shows steady-state analysis of equilibrium response (R_eq_) plotted against C7 concentration and fitted to a single-site binding isotherm to derive the equilibrium dissociation constant. Response is reported as wavelength shift (nm). (**D**) C7 inhibits irreversible structural transitions of apo-SOD1^WT^. Apo-SOD1^WT^ (50 μM) was incubated with or without C7 (100 μM; 1% DMSO) at 37 °C under misfolding-promoting conditions. Protein samples were withdrawn at the indicated time points and incubated for 2 h at room temperature with ZnCl_2_ (100 μM). Following incubation, the ability of SOD1 to bind SE21 mAb was assessed by ELISA as described in Methods. Results represent mean ± SD and are representative of at least three independent experiments performed in triplicate.

The compounds selected by the VS were tested for their ability to inhibit SE21 mAb binding to apo-SOD1, either WT or fALS-associated SOD1^G93A^ mutant, using ELISA. One compound, referred to as C7 (Fig. 1B), suppressed SE21 mAb binding to both SOD1 variants (Fig. 2A), exhibited a dose-dependent inhibition with a non-zero saturation plateau (Fig. 2A, inset). Based on its physicochemical properties, C7 was selected for further functional characterization. Importantly, C7 did not inhibit binding of a polyclonal anti-SOD1 antibody, which does not discriminate between intact and misfolded SOD1 conformers, to apo-SOD1, demonstrating that C7 binding selectively affected the accessibility of the pathogenic β6/β7 loop neo-epitope (Fig. 2A).

To quantitatively characterize C7 binding to SOD1, we used microscale thermophoresis (MST), demonstrating that C7 binds holo-SOD1^WT^ with significantly higher affinity (logIC_50_ = −5.20 ± 0.067, IC_50_ = 6.32 μM) than apo-SOD1^G93A^ (approximated IC_50_ ≈ 96 μM) (Fig. 2B). Given that structural destabilization of SOD1 increases the probability of misfolding [10,11,[23], [24], [25]], a compound that preferentially binds and stabilizes SOD1 in a native-like (i.e., holo-SOD1^WT^-like) conformation would be expected to suppress misfolding. We further analyzed C7 binding kinetics to holo-SOD1^WT^ using Octet biolayer interferometry (BLI). Global kinetic fitting of reference-subtracted BLI sensorgrams yielded a micromolar-affinity interaction, with k_on_ ≈ 2.4 × 10^3^ M^−1^ s^−1^ and k_off_ ≈ 2.1 × 10^−3^ s^−1^ (Kd ≈ 0.9 μM) (Fig. 2C). Independent steady-state analysis gave a comparable affinity estimate (Kd ≈ 1.2 μM), supporting the validity of the kinetic model (Fig. 2C, inset). Binding of C7 to apo-SOD1^G93A^ was too weak to permit reliable quantitation by this assay (not shown). These findings are consistent with preferential binding of C7 to a native-like SOD1 conformation. The stabilizing effect of binding may inhibit the transition of SOD1 into a misfolded state.

SOD1 noxious structural transformation (misfolding) is a multistep process facilitated by SOD1 demetalation, which results in the formation of atypical apo-SOD1 species deficient in their ability to bind metal cofactors [16,26]. We have previously demonstrated that, in intact SOD1 proteins, metal loss leads to a reversible exposure of the β6/β7 loop epitope, followed by an irreversible structural transition that generates soluble misfolded SOD1 species unable to rebind metal cofactors and thus exhibiting permanently exposed β6/β7 loop epitopes [16]. C7 demonstrated the ability to inhibit the kinetics of this irreversible structural transition of apo-SOD1^WT^ incubated under misfolding-promoting conditions (Fig. 2D), supporting the notion of its stabilizing effect on SOD1 structure.

According to the binding mode generated by VS docking, C7 is predicted to bind within the surface pocket shaped by the two parallel β6/β7 loops of the SOD1 dimer and to form electrostatic interactions simultaneously with both Arg115 residues from the two subunits (Fig. 1C and D and Supplementary Fig. S1B). Arg115 is located within the Greek-key loop VI of SOD1 and participates in inter-subunit interactions that contribute to stabilization of the dimer interface [27]. Mutations at this residue have been shown to cause fALS [28,29]. It is therefore tempting to speculate that crosslinking of the two Arg115 residues by C7 at the dimer interface may exert an additional stabilizing effect on the dimer interface and the overall SOD1 fold.

### Direct ‘nose-to-brain’ spray delivery of C7 ameliorates disease phenotype in hSOD1^G93A^ ALS mice

To test whether exposure to C7 can alter the disease course in an animal model of ALS, we used a nanoparticle (NP)-based *nose-to-brain* delivery system to administer C7 into the CNS of mice transgenic for the human SOD1^G93A^ variant. This NP system has been shown to facilitate CNS delivery of multiple active compounds with diverse physicochemical properties (e.g., lipophilicity, molecular weight) [20,30]. Beyond its ability to bypass the BBB, intranasal administration provides a non-invasive and direct route to the brain, enabling rapid delivery with minimal systemic exposure and thereby reducing the risk of peripheral side effects. It also avoids first-pass hepatic metabolism, enhancing bioavailability and potentially lowering the required dosage [31]. Moreover, intranasal delivery is patient-friendly, offering a convenient alternative to injections or oral medications, which may improve adherence in chronic neurodegenerative conditions.

**Fig. 3:**
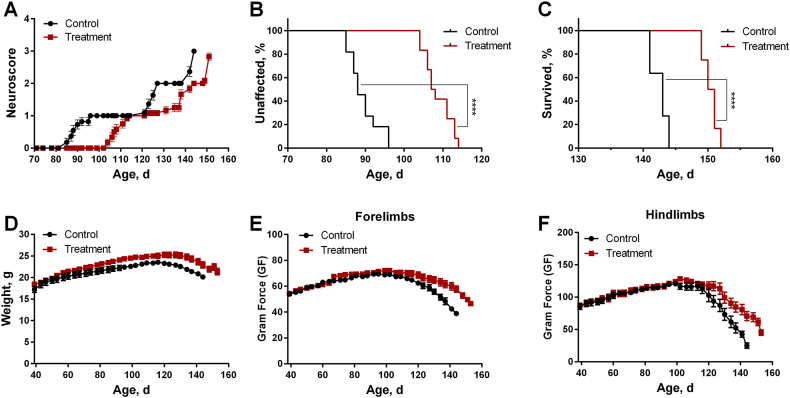
**Intranasal C7 treatment delays disease onset and prolongs survival in SOD1^G93A^ ALS mice (males).** (**A**) Disease progression assessed by NeuroScore (NS). Average NS was calculated for mice of the same age within each cohort and plotted as a function of age. (**B**) Kaplan–Meier analysis of disease onset (percent unaffected), defined as the age at which the first motor abnormalities appeared (NS = 1). (**C**) Kaplan–Meier analysis of survival (percent survived), defined as progression to paralysis (NS = 3). (**D**) Body weight. (**E**) Forelimb grip strength. (**F**) Hindlimb grip strength. Statistical significance in Kaplan–Meier analyses was assessed using the Log-rank (Mantel–Cox) test. Data in panels A and D–F are presented as mean ± SEM. Sample sizes were n = 12 for C7-treated mice and n = 11 for control mice. ∗∗∗∗p < 0.0001.

A cohort of 30 gender-balanced SOD1^G93A^ mice was treated daily with C7-loaded NP Formulation 2 (30 μg, see the Methods) by intranasal administration (IN) starting at 25–28 days of age, while control animals received an empty NP formulation. C7 treatment significantly delayed the onset of motor abnormalities (NS = 1) in both sexes. In male mice, median onset was extended from 88.0 to 107.5 days (log-rank test, p < 0.0001), corresponding to an approximately 30-fold reduction in onset risk (Mantel–Haenszel HR = 30.57, 95% CI: 8.05–116.1; Fig. 3A and B). A comparable effect was observed in females, in which median onset was delayed from 86.5 to 109.5 days (log-rank test, p < 0.0001) and onset risk was reduced by ∼25-fold (HR = 25.26, 95% CI: 6.38–100.0; Supplementary Fig. S2A and B).

Beyond delaying disease onset, C7 treatment also conferred a modest but statistically significant improvement in overall survival, defined as progression to paralysis (NS = 3). In treated males, median survival increased from 143.0 to 150.5 days (log-rank test, p < 0.0001), with a corresponding reduction in mortality risk (HR = 30.65, 95% CI: 7.80–120.5; Fig. 3A–C). Similarly, in treated females, median survival was extended from 144.0 to 151.5 days (log-rank test, p < 0.0001), with a corresponding reduction in mortality risk (HR = 22.49, 95% CI: 5.59–90.42; Supplementary Fig. S2A and C). Other disease markers, including loss of body weight and grip strength, showed a consistent trend toward improvement in C7-treated animals (Fig. 3D–F and Supplementary Fig. S2D–F). Administration of the empty nanoparticle (NP) formulation (control animals) had no significant effect on behavioral disease markers compared with untreated animals (Supplementary Fig. S3).

**Fig. 4:**
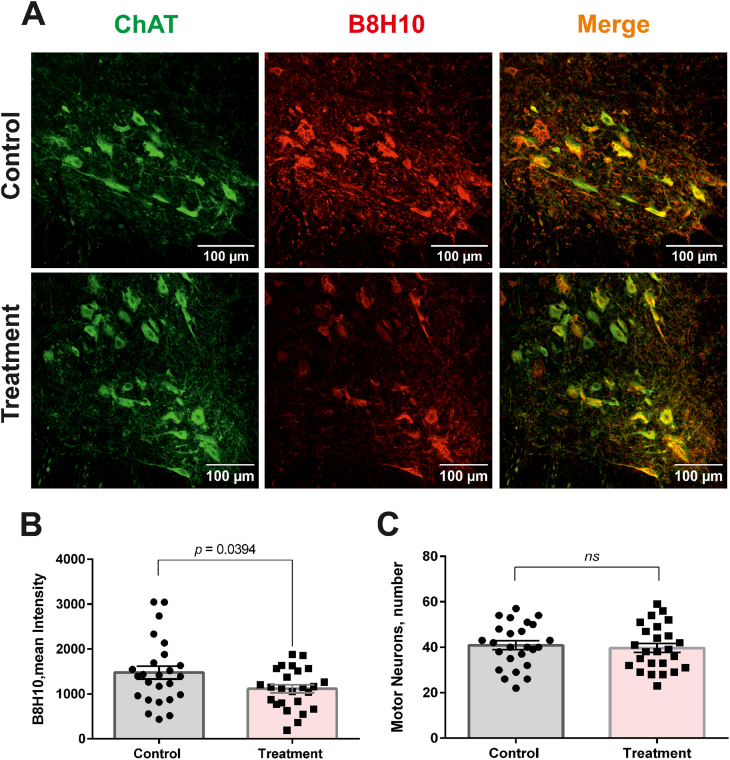
**Intranasal administration of C7 reduces spinal accumulation of misfolded SOD1 in SOD1^G93A^ mice (females).** (**A**) Representative fluorescence micrographs of lumbar spinal cord ventral horn sections from SOD1^G93A^ mice, immunostained with anti-ChAT to label motoneurons (green) and with the conformation-specific antibody B8H10 to detect aggregated misfolded SOD1 (red). (**B**) Quantitative assessment of misfolded SOD1 burden in the lumbar spinal cord corresponding to panel (A). Analysis was conducted as described in the Methods by measuring mean fluorescence intensity within defined ROIs. Data represent mean ± SEM derived from 25 sections obtained from five mice per group (n = 5). Statistical significance was determined using an unpaired parametric *t*-test. (**C**) Quantification of ChAT-positive motoneurons in the lumbar spinal cord corresponding to the sections shown in panel (A). Motoneuron counts were performed as detailed in the Methods. Data are presented as mean ± SEM based on analysis of 25 sections from five mice per group (n = 5). Group differences were evaluated using an unpaired parametric *t*-test.

**Fig. 5:**
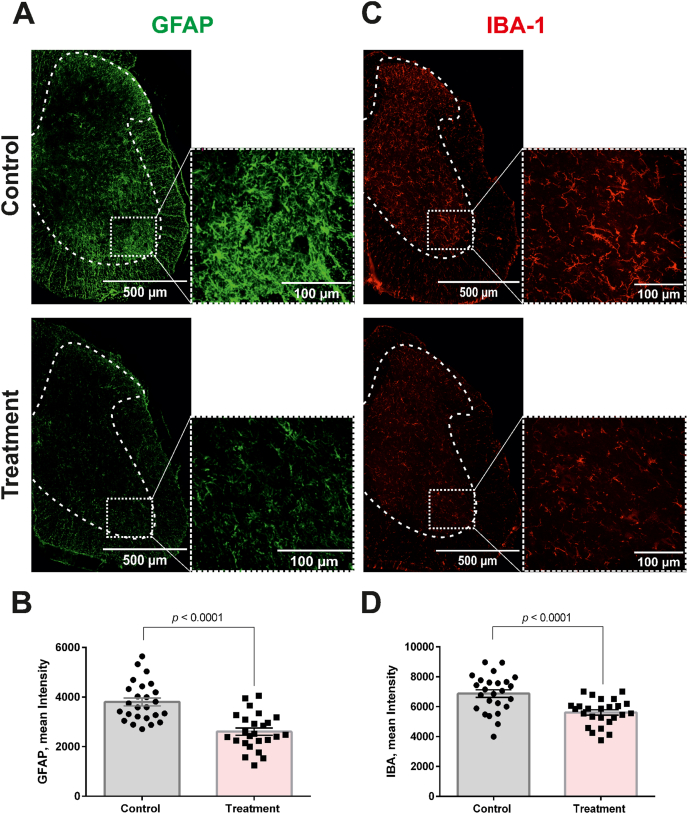
**Intranasal delivery of C7 attenuates astrocytic and microglial reactivity in the spinal cord of SOD1^G93A^ mice (females).** Representative immunofluorescence images of lumbar spinal cord ventral horn sections from SOD1^G93A^ mice showing GFAP immunoreactivity (**A**) as a marker of reactive astrocytes and Iba1 immunoreactivity (**C**) as a marker of activated microglia. Quantification of astrocyte (**B**) and microglial (**D**) activation corresponding to panels A and C, respectively, was carried out as detailed in the Methods section. All results are expressed as mean ± SEM of fluorescence intensity within the defined region of interest (ROI), averaged from 25 spinal cord sections derived from five mice per group (n = 5). Group differences were evaluated using an unpaired two-tailed *t*-test.

Molecular pathology analysis of spinal cord tissue performed at 90 days of age—corresponding to disease onset in the control group—demonstrated improved markers of disease-associated pathology. Immunostaining with the B8H10 antibody, which recognizes misfolded conformations of SOD1 [32], revealed a decreased accumulation of misfolded SOD1 inclusions in the spinal cord (Fig. 4A and B). Treated animals also displayed significantly reduced neuroinflammatory histopathology compared with controls, as evidenced by decreased GFAP and Iba1 immunoreactivity in the lumbar spinal cord gray matter (Fig. 5A–D), consistent with reduced astroglial and microglial reactivity, respectively. However, because these measurements were based on mean fluorescence intensity, they do not by themselves distinguish among changes in glial cell number, morphology, or specific activation state.

In contrast, quantification of ChAT-positive motor neurons in the spinal cord at this early disease stage revealed no significant difference between the treated and control groups (Fig. 4A–C). Because ChAT-positive motor neurons were quantified using a non-stereological section-based method, the absence of a detectable group difference at 90 days should be interpreted with caution and not as a definitive absence of subtle neuroprotective effects.

### C7 pharmacokinetics is consistent with saturable uptake and slow CNS clearance

We measured C7 concentrations in combined brain and spinal cord tissue from SOD1^G93A^ mice treated with two different nanoparticle formulations and cumulative dosing regimens. In the first paradigm, 40-day-old mice received intranasal (IN) C7 twice daily for two weeks at a total dose of 2.8 μg per mouse per day, using a previously reported ALN nanoparticle (NP) formulation [20,30] that allowed only limited C7 loading (140 μg/mL; Formulation 1, see Methods). Mice were sacrificed 1 h after the final administration, and C7 levels in combined brain and spinal cord tissue were determined as described in the Methods.

In the second paradigm, 25-day-old mice received IN C7 once daily for over three months at a total dose of 30 μg per mouse per day, using a modified NP formulation that enabled substantially higher C7 loading (3 mg/mL; Formulation 2, see Methods). Mice were sacrificed at 120 days of age, 1, 3, or 6 h after the final administration, and C7 concentrations in CNS tissue were analyzed.

The results summarized in Table 1 demonstrate rapid penetration and nonlinear pharmacokinetics of C7 in the CNS: similar high-ng/g levels (210–680 ng/g dry tissue) were observed after a low cumulative dose administered over two weeks and after a substantially higher daily dose administered for 3 months, suggesting that nose-to-brain transport and/or CNS uptake of C7 is saturable. In addition, after chronic administration, C7 remained detectable in the CNS at high-ng/g levels for at least 6 h after the final dose, without a consistent monotonic decline across the sampled time points (1–6 h). These data are consistent with slow elimination and/or sustained release of C7 from nasal and/or CNS depots. Moreover, considerable mouse-to-mouse variability in CNS exposure was observed, likely reflecting anatomical and/or metabolic inter-animal differences that affect nasal deposition of C7 and/or its mucociliary clearance [33].

**Table 1:** **Intranasally delivered C7 demonstrates nonlinear pharmacokinetics in the CNS.** SOD1^G93A^ mice were exposed to C7 using two different formulations and dosing regimens (as described in the text). C7 levels in combined brain and spinal cord tissue were measured at the indicated time points after the final administration, as described in the Methods. Data are presented as mean ± SD.

NP Formulation	Daily dose, (μg/mouse)	Gender	Time after final administration	C7 concentration in CNS (μg/g dry tissue)
1	2.8	Male	1 h	0.68 ± 0.63 (n = 4)
2	30	Female	1 h	0.57 ± 0.30 (n = 3)
2	30	Female	3 h	0.21 ± 0.26 (n = 3)
2	30	Female	6 h	0.44 ± 0.16 (n = 3)
2	30	Male	3 h	0.34 ± 0.18 (n = 3)

The clinical efficacy experiments in SOD1^G93A^ mice demonstrated that both NP formulations and dosing regimens, namely 2.8 and 30 μg C7/day, were similarly effective in delaying disease onset (compare Fig. 3A and B with Supplementary Fig. S4), consistent with the comparable CNS exposure to C7 achieved under both conditions. The saturable nature of nose-to-brain uptake likely imposes an upper limit on achievable CNS exposure and thus potentially restricts the therapeutic efficacy of C7. Defining the pharmacokinetic bottleneck(s) that constrain CNS bioavailability will be essential for advancing this lead and may require optimization of the nanoparticle formulation and/or the physicochemical properties of the C7 scaffold.

As described in the Introduction, the overarching hypothesis underlying this drug discovery effort was that preventing the early steps of noxious SOD1 structural transformation could be achieved by blocking the exposed pathogenic β6/β7 loop neo-epitope through binding of a small-molecule compound to the surface cleft formed by the two parallel β6/β7 loops in the intact SOD1 dimer. Such binding was expected to sterically interfere with the insertion of the exposed β6/β7 loop from another misfolded SOD1 molecule, thereby preventing the formation of the aberrant trimeric complex, a proposed milestone in the mechanism of prion-like spreading of misfolded SOD1 pathology [15]. The mechanism by which C7 exerts its therapeutic efficacy is currently unknown; however, in addition to the steric interference with the formation of abnormal SOD1 complexes described above, the data presented here indicate the involvement of another, although mutually nonexclusive, mechanism. Specifically, by bridging the SOD1 dimer at its interface, C7 may exert an overall stabilizing effect on the SOD1 fold, which, in addition to concealing the β6/β7 loop epitope, may prevent structural transition into a thermodynamically coupled misfolded state [10,11,[23], [24], [25]]. A similar idea has been explored in previous work, in which stabilization of the SOD1 interface was attempted by bridging two solvent-exposed Cys111 residues, which face the cavity formed by the two β6/β7 loops, using cysteine-reactive small molecules that chemically modify Cys111 [[34], [35], [36], [37], [38]]. Here, we demonstrate that a meaningful therapeutic effect can be achieved by a reversibly binding small-molecule compound, warranting further studies in this direction.

The present findings should also be considered in the context of our previous β6/β7-loop antibody-based study. In that work, AAV-mediated neuronal expression of the intrabody scFv-SE21 in hSOD1^G37R^ mice delayed disease onset by 94 days and extended survival by 89 days, a substantially larger effect than that observed here with C7. However, direct comparison between the two studies warrants caution, as they differ in SOD1 model, disease aggressiveness, route of delivery, and treatment paradigm. The intrabody was expressed continuously from birth and acted intracellularly, primarily in neurons, whereas C7 was administered intranasally as a free pharmacological agent and achieved CNS exposure that appears saturable and variable. These differences likely contribute to the lower apparent *in vivo* efficacy of C7. Thus, the present study should be viewed as a proof-of-concept extending the earlier work by showing that a structural determinant previously validated by antibody-based targeting is also chemically druggable and can be engaged by a non-covalent small molecule, thereby providing a more conventional therapeutic-development route that is amenable to medicinal-chemistry and formulation optimization.

As with our previous antibody-based study, an important limitation of the present work is that C7 treatment was initiated at a presymptomatic stage. Although advances in genetic screening have begun to shift the therapeutic paradigm in familial ALS toward prophylactic strategies, the current design is primarily suited to testing the biological importance and draggability of the β6/β7-loop structural axis and does not fully reflect the clinical setting, in which most ALS patients begin treatment only after symptom onset. The current results therefore define C7 as a lead compound capable of modifying early pathogenic events *in vivo*, whereas its therapeutic window remains to be established.

## Ethics approval and consent to participate

All animal procedures were approved by the Ben-Gurion University Animal Care and Use Committee (IACUC) and the Israel National Council for Animal Experimentation (protocol # BGU322-05-2024E).

## Availability of data and materials

The data supporting the findings of this study are available within the paper and its Supplementary Information. Additional raw datasets are available from the corresponding author upon request.

## Authors contributions

Conceptualization, S.E., A.S.**¶**; Methodology, S.E., A.S.**¶**; Investigation & Analysis, R.D., G.Y., S.B., A.A., M.B., A.S.∗, A.S**¶**., A.T.; Writing – Original Draft, S.E.; Writing – Revision & Editing, S.E., A.S**¶**, R.D., Z.B.; Resources, S.E., A.S**¶**, Z.B.; Supervision, S.E. All authors provided critical feedback and helped shape the research, analysis and manuscript.

## Declaration of generative AI and AI-assisted technologies in the manuscript preparation process

During the preparation of this work the author(s) used ChatGPT 5.2 tool for proofreading the final text, with only minimal and absolutely necessary changes accepted. The author(s) take(s) full responsibility for the content of the published article.

## Funding

This work was partially supported by 221/22 grant from the 10.13039/501100003977Israel Science Foundation.

## Declaration of competing interests

The authors declare that they have no conflict of interests with the contents of this article.
