# Supplementary material for: Nose-to-brain delivery of a SOD1-stabilizing small molecule ameliorates pathology in an ALS mouse model
Source: Neurotherapeutics. 2026 Apr 10;23(3):e00904. doi: 10.1016/j.neurot.2026.e00904 (PMC13092042; doi:10.1016/j.neurot.2026.e00904)
Supplement: Multimedia component 1 [file mmc1.pdf]

Supplementary Figures S1-S4.

Nose-to-brain delivery of a SOD1-stabilizing small molecule ameliorates pathology in an ALS mouse model. Ranjithkumar Dhandapani, *et.al*

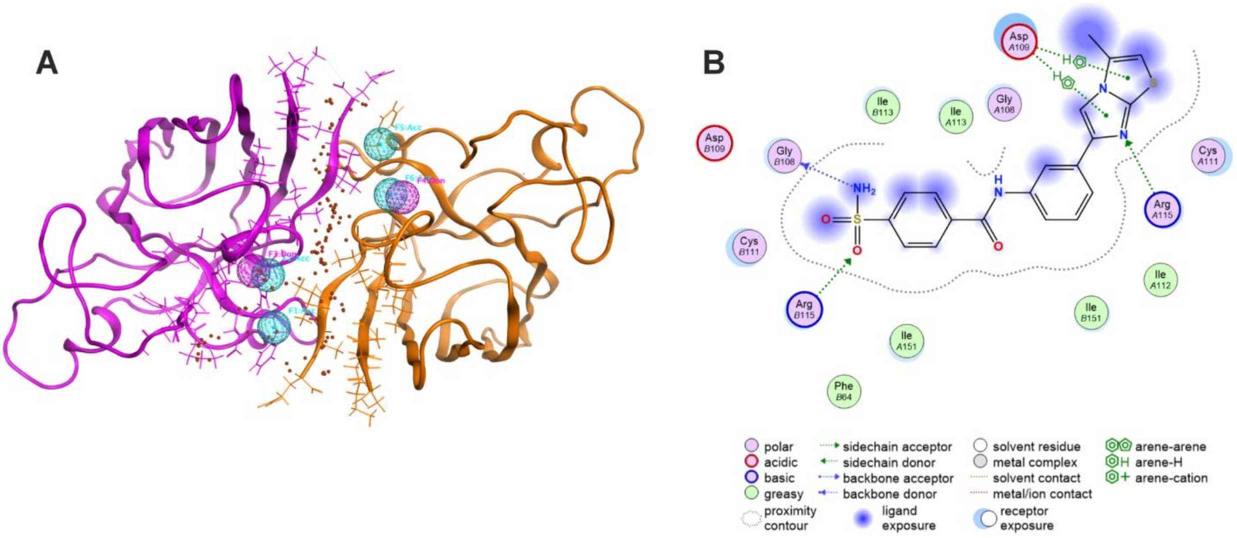

Supplementary Figure S1. **Identification of the SOD1 dimer-interface binding pocket and predicted interactions of the lead compound C7 within this site.** (**A**) 3D pharmacophore model of the inter-subunit binding site on SOD1 (template structure: PDB 1HL5). Cartoon representation of the Cu/Zn-SOD1 homodimer, with one subunit colored magenta and the other orange. The putative small-molecule binding pocket is located in the interfacial cleft between the two parallel β6/β7 loops, as identified by MOE Site Finder (SITE 1, see the *Methods*). Protein side chains lining the pocket are shown in line representation, and the cavity volume is outlined by dummy atoms, which serve as matching points for ligand docking. The 3D pharmacophore derived from this pocket is depicted as wire-mesh spheres: hydrogen-bond acceptors are shown in cyan (features F1, F2, F5 and F6), and hydrogen-bond donors in magenta (features F3 and F4). (**B**) Two-dimensional ligand–protein interaction diagram generated in MOE for compound C7 docked into the inter-subunit cleft of the Cu/Zn-SOD1 homodimer. The C7 binding pocket constitutes a predominantly hydrophobic groove delineated by Ile^{112}, Ile^{113}, and Cys^{111} from both subunits, with discrete polar “hot spots” that stabilize ligand binding across the dimer interface. C7 is predicted to form hydrogen-bond interactions (green dashed arrows) with Arg^{115} from subunit A via its heteroaromatic headgroup and with Arg^{115} from subunit B via its sulfonamide terminus, thereby bridging the two SOD1 subunits within the dimer-interface pocket.

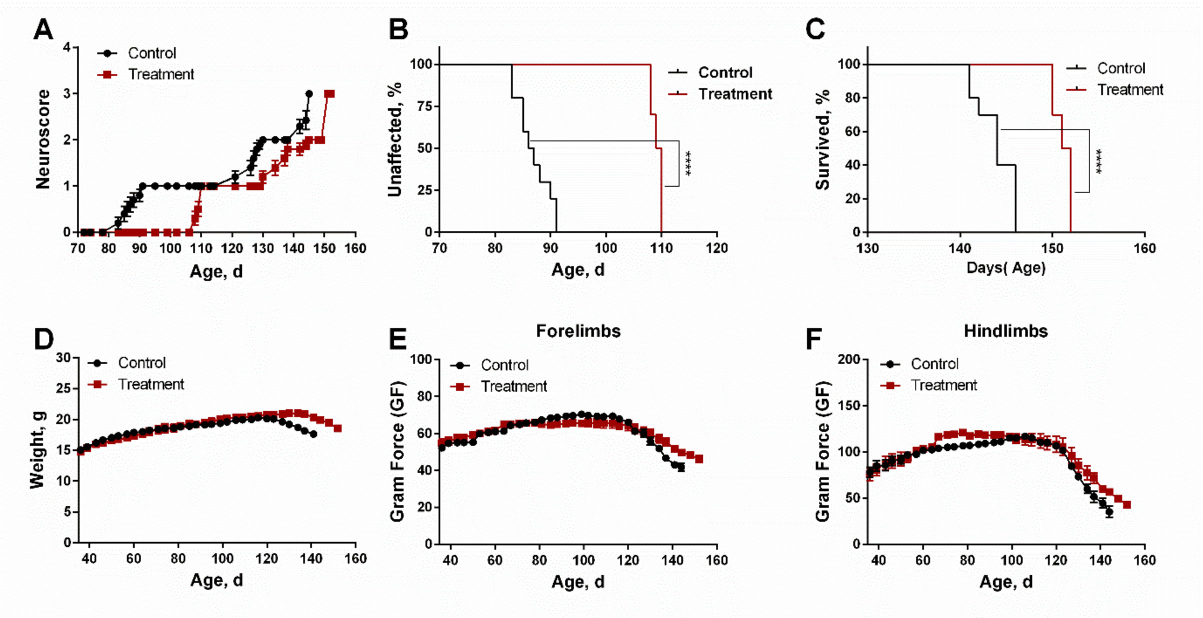

Supplementary Figure S2. Intranasal C7 treatment delays disease onset and prolongs survival in SOD1^{G93A} ALS mice (females). (**A**) Disease progression assessed by NeuroScore (NS). Average NS was calculated for mice of the same age within each cohort and plotted as a function of age. (**B**) Kaplan–Meier analysis of disease onset (percent unaffected), defined as the age at which the first motor abnormalities appeared (NS = 1). (**C**) Kaplan–Meier analysis of survival (percent survived), defined as progression to paralysis (NS = 3). (**D**) Body weight. (**E**) Forelimb grip strength. (**F**) Hindlimb grip strength. Statistical significance in Kaplan–Meier analyses was assessed using the Log-rank (Mantel–Cox) test. Data in panels A and D–F are presented as mean ± SEM. Sample sizes were n = 10 for C7-treated mice and n = 10 for control mice. **** $p < 0.0001$.

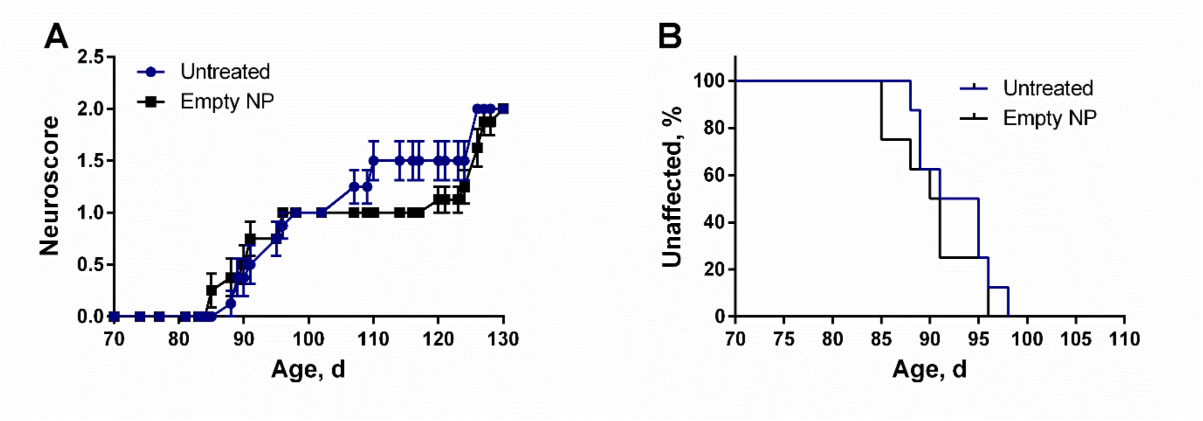

Supplementary Figure S3. Intranasal administration of empty NP formulation does not affect disease course in SOD1^{G93A} ALS mice. (**A**) Disease progression assessed by NeuroScore (NS). Average NS was calculated for mice of the same age within each cohort and plotted as a function of age. (**B**) Kaplan–Meier analysis of disease onset (percent unaffected), defined as the age at which the first motor abnormalities appeared (NS = 1). No statistically significant difference was detected between the two groups by the log-rank (Mantel–Cox) test. Sample sizes were n = 8 for mice administered with empty NP and n = 8 for untreated mice.

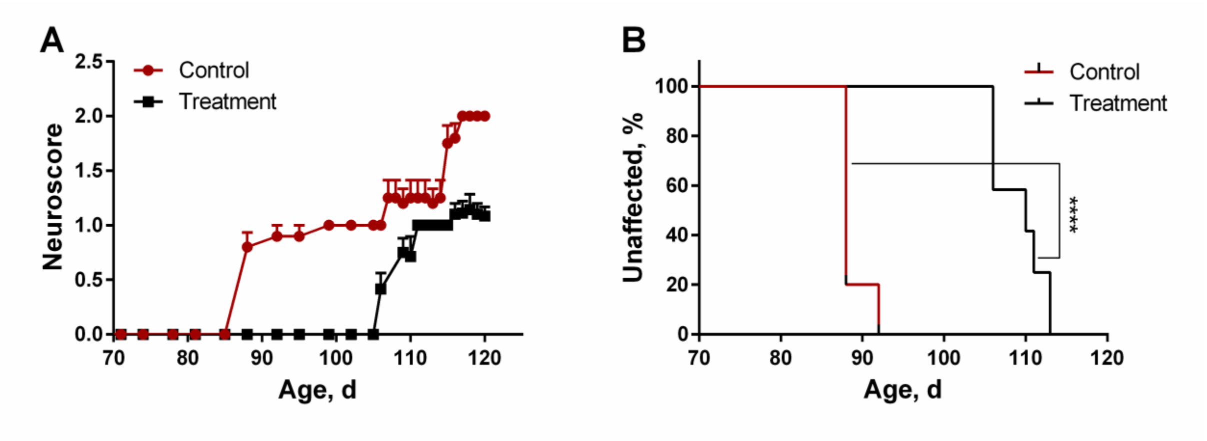

Supplementary Figure S4. CNS exposure to C7 correlates with disease modification in SOD1^{G93A} ALS mice. Twenty-five-day-old sex-balanced SOD1^{G93A} mice were administered C7 intranasally twice daily at a total dose of 2.8 µg/day using a previously described ALN nanoparticle (NP) formulation with limited C7 loading capacity (~150 µg/ml). Control animals received the empty ALN NP formulation. (**A**) Disease progression assessed by NeuroScore (NS). (**B**) Kaplan–Meier analysis of disease onset (percent unaffected), defined as the age at which the first motor abnormalities appeared (NS = 1). C7 treatment significantly delayed disease onset compared with controls (Log-rank [Mantel–Cox] test, $p < 0.0001$). Median onset was extended from 88.0 days in control mice to 110.0 days in treated animals, corresponding to a significant reduction in onset risk (Mantel–Haenszel hazard ratio = 50.67, 95% CI: 10.49–244.8). Group sizes were n = 12 for C7-treated mice and n = 10 for empty NP controls, with males and females balanced across groups. **** $p < 0.0001$.
